# Recent Trends in Biofabrication Technologies for Studying Skeletal Muscle Tissue-Related Diseases

**DOI:** 10.3389/fbioe.2021.782333

**Published:** 2021-10-27

**Authors:** Seungyeun Cho, Jinah Jang

**Affiliations:** ^1^ Department of Convergence IT Engineering, Pohang University of Science and Technology, Pohang, South Korea; ^2^ School of Interdisciplinary Bioscience and Bioengineering, Pohang University of Science and Technology, Pohang, South Korea; ^3^ Department of Mechanical Engineering, Pohang University of Science and Technology, Pohang, South Korea; ^4^ Institute for Convergence Research and Education in Advanced Technology, Yonsei University, Seoul, South Korea

**Keywords:** disease modelling, hiPSC, extrusion printing, volumetric muscle loss, muscular dystrophy, self-repair

## Abstract

In native skeletal muscle, densely packed myofibers exist in close contact with surrounding motor neurons and blood vessels, which are embedded in the fibrous connective tissue. In comparison to conventional two-dimensional (2D) cultures, the three-dimensional (3D) engineered skeletal muscle models allow structural and mechanical resemblance with native skeletal muscle tissue by providing geometric confinement and physiological matrix stiffness to the cells. In addition, various external stimuli applied to these models enhance muscle maturation along with cell–cell and cell–extracellular matrix interaction. Therefore, 3D *in vitro* muscle models can adequately recapitulate the pathophysiologic events occurring in tissue–tissue interfaces inside the native skeletal muscle such as neuromuscular junction. Moreover, 3D muscle models can induce pathological phenotype of human muscle dystrophies such as Duchenne muscular dystrophy by incorporating patient-derived induced pluripotent stem cells and human primary cells. In this review, we discuss the current biofabrication technologies for modeling various skeletal muscle tissue-related diseases (i.e., muscle diseases) including muscular dystrophies and inflammatory muscle diseases. In particular, these approaches would enable the discovery of novel phenotypic markers and the mechanism study of human muscle diseases with genetic mutations.

## Introduction

Skeletal muscles constitute 35–40% of the human body weight and are essential for maintaining the posture, locomotion, and respiration of the human body. The tissue microenvironment comprises uniaxially aligned multinucleated muscle cells (myofibers) that are arranged in a three-dimensional (3D) extracellular matrix (ECM) scaffold. Owing to the presence of muscle-resident stem cells, also referred as satellite cells (SCs), the healthy skeletal muscles exhibit an inherent capacity for regeneration in response to small-scale injuries. However, the muscular self-repair mechanism is hindered by traumatic or surgical injuries, genetic mutations in ECM and sarcolemmal proteins, neurodegenerative diseases, aging-induced alterations in ECM compositions, regulatory cell behaviors, and signaling pathways that result in a wide range of diseases (Relaix et al., 2021).

In context, Duchenne muscular dystrophy (DMD) is a notable progressive muscle-wasting disease that results from failed production of dystrophin—a protein that stabilizes the myofibers. Although several antisense oligonucleotide-mediated exon-skipping therapies including eteplirsen, golodirsen, and viltolarsen treatment were granted conditional approval by the United States Food and Drug Administration (FDA), no current cure exists for the DMD (Bursac et al., 2015; Alfano et al., 2019; Clemens et al., 2020; Frank et al., 2020). Charcot–Marie–Tooth disease (CMT) is the most commonly inherited neuromuscular disorder that causes distal muscle atrophy and results in a high-arched foot, impaired gait, and frequent falls. Although PXT3003—a drug that downregulates PMP22 mRNA expression—entered phase III of clinical trials in 2021, no clinically approved therapies currently exist for CMT (Vallat et al., 2013).

Genetically engineered mice lacking dystrophin and/or utrophin (a homolog of dystrophin) have been utilized to study the mechanism of DMD. Dystrophin-deficient mdx mice recapitulate mild dystrophic phenotypes. Compared with dog models and affected humans, mdx mice cannot model more severe disease phenotypes such as limb muscle fibrosis (McGreevy et al., 2015). However, dystrophin–utrophin double knockout (dko) mice (i.e., dko model) have been suggested as a more useful model to study the disease, as utrophin can compensate for the absence of dystrophin (Grady et al., 1997). Notably, a wide spectrum of degenerative musculoskeletal abnormalities has been reported using dko model (Isaac et al., 2012). However, this additional mutation in another gene, excluding dystrophin, does not correlate with the case in affected humans (McGreevy et al., 2015).

This genetic discrepancy has adversely affected the correct prediction of drug responses, as exemplified by the market withdrawal of cerivastatin, which caused fatal myopathies in human but was well-tolerated in mice (Furberg and Pitt et al., 2001). Although Idebenone apparently reduced the cardiac inflammation and fibrosis in a preclinical study with mdx mice, the phase III of the clinical trials assessing its capacity on delaying the respiratory decline in DMD patients was ceased in October 2020 after failing an interim analysis (Buyse et al., 2009). Thus, a preclinical human muscle tissue-related disease model is required to be developed to accurately mimic human pathophenotypes and disease progressions.

In context, complex biomimetic skeletal muscle structures have been developed using numerous biofabrication technologies that support the growth and maturation of cells in a 3D arrangement. Lithography-based methods and microfluidic devices have been utilized on micropattern surfaces with aligned topographical features (e.g., grooves) onto which seeded cells were directed to uniaxially align and differentiate into myofiber-like structures. Recent advancements in biofabrication methods such as 3D printing technology and electrospinning have yielded 3D tissue-engineered muscle models with multiscale structural cues resembling the hierarchical tissue geometry.

Physiologically relevant skeletal muscle phenotypes were obtained by placing endothelial, stromal, and neural cells in contact with myofibers, which could more precisely mimic the paracrine interactions occurring in the native tissue. In addition, the cell–matrix interaction that influences force transmission in native myofibers were also modeled. By contrast, conventional 2D models such as monoculture cellular assays are limited in recapitulating complex tissue architectures such as hierarchical arrangement. In addition, 2D models have limited capacity for muscle maturation, as contracting myofibers detach from the substrate, whereas 3D models support spontaneous cell fusion and myofiber enlargement (which resembles muscle hypertrophy) throughout several weeks of culture. Based on these advantages of the 3D models, a wide range of human muscle diseases have been recapitulated “in a dish.” In addition, the incorporation of human-induced pluripotent stem cells (hiPSCs) enables the recapitulation of patient-specific disease phenotypes in these models.

In this review, we discuss the biomaterials widely utilized as 3D scaffold materials for muscle cells, followed by a review of multiple biofabrication technologies that yield aligned myofibers within the aforementioned biomaterials, such as shear/tensile force, aligned geometrical cues, and 3D bioprinting skills that allow deposition of cell-laden hydrogels in a desired geometry. Subsequently, we address the recent examples of vascularization, innervation, and electrical/mechanical stimuli that allowed additional maturation of 3D-engineered muscle. Ultimately, we highlight the application of these techniques for human muscle tissue-related disease modeling (i.e., muscular dystrophies and inflammatory muscle diseases).

## Biomaterials Utilized in 3D Tissue-Engineered Muscle

In conventional 2D cultures, the mechanical properties of the rigid tissue culture substrates do not correspond to those of the native skeletal muscle tissue, which results in developmentally immature myotube structures such as poor striations and premyofibril myosin organization (Engler et al., 2004). Instead, several natural polymers have been proposed as a scaffold material to culture muscle cells in a 3D environment. In this section, we reviewed multiple biomaterials that are frequently used in fabricating 3D-engineered muscle, such as collagen, fibrin, and decellularized extracellular matrix (dECM) (Table 1). In addition, Matrigel, which is a basement membrane extract originating from Engelbreth–Holm–Swarm (EHS) sarcoma and is rich in ECM proteins such as laminin and collagen IV, has been used in combination with collagen and fibrin.

**TABLE 1:** Different types of biomaterials used in skeletal muscle tissue engineering.

Biomaterials	Pros/Cons	Achievement	Relevant bioactivities	References
Collagen	Structural cues/low remodeling capacity	Uniaxial cell alignment	Fibril bundle formation	Kim and Kim et al. (2019)
		Long-term construct stability	Cell attachment	Morimoto et al. (2020)
			Large pore size	
Fibrin	High contractile force/batch-to-batch variability	Human fetus-relevant force production	Native tissue stiffness	Hinds et al. (2011)
		High myofiber and vascular density after implantation	Pro-angiogenic effect	Gilbert-Honick et al. (2018)
Laminin	Cell attachment/degradation	Dose-dependent increase in cell proliferation	α7β1 integrin expression	Marcinczyk et al. (2017)
dECM	Biochemical and structural cues/low structural stability	*In vitro* AChR cluster formation	Presence of agrin	Choi et al. (2016)

Collagen is an abundant component of the ECM in native muscle tissue that was utilized in the earliest cases of 3D-engineered muscle (Powell et al., 2002). Powell et al. (2002) observed that human primary muscle cells embedded in collagen/Matrigel mixture formed parallel arrays of differentiated myofibers under passive tension. However, the absence of collagen I-specific integrin in myotubes resulted in lesser contractility than that of the other types of integrin-binding ECM proteins such as fibrin and laminin (Mayer, 2003). Therefore, increasing the concentration of collagen could adversely affect the force generation (Hinds et al., 2011). In another research, Dixon et al. (2018) prepared a collagen/Matrigel/silk hydrogel that successfully induced myogenic differentiation, maintained its mechanical integrity during the compacting around silk cantilevers, and formed a 3D-engineered muscle (Dixon et al., 2018).

Fibrin is the fundamental component of a blood clot that is formed in the initial steps of the wound-healing process, and subsequently, replaced by the cell-produced ECM. Upon using in skeletal muscle tissue engineering, fibrin can exert a passive stiffness that is several orders of magnitude lower than that of collagen and comparable to that of native skeletal muscle. Madden et al. (2015) demonstrated tetanus force production that corresponded to that of the fetal human skeletal muscle in a fibrin-based 3D-engineered muscle. In addition, fibrin often prevents tissue deformation under tissue remodeling owing to its capacity to be degraded and replaced by endogenous ECM. Christensen et al. (2020) fabricated a contractile muscle tissue with fibrin/Matrigel, which was anchored to two cantilevers and maintained the tissue confined during cell-mediated compaction. Thus, the fibrin can support the spontaneous formation of contractile myotubes (Madden et al., 2015; Christensen et al., 2020).

Lastly, dECM has emerged as a potential candidate for inducing the elongation and fusion of muscle cells owing to its tissue-specific cell-binding moieties, ECM topography, and physical ultrastructures. Furthermore, studies have demonstrated that dECM could induce tissue-specific cell differentiation (Flynn, 2010; Uygun et al., 2010). Choi et al. (2016) developed the preparation process of skeletal muscle decellularized extracellular matrix (mdECM). Notably, agrin—an ECM protein that enhances the clustering of acetylcholine receptors—was conserved in the mdECM (Choi et al., 2016). In addition, dECM can be chemically processed or blended with other polymers before electrospinning or extrusion printing to fabricate a 3D scaffold integrated with tissue-specific biochemical cues and versatile structural cues (Baiguera et al., 2014; Kim et al., 2020; Kim et al., 2021). Baiguera et al. (2014) blended rat brain-derived dECM with gelatin prior to electrospinning and obtained a highly porous 3D scaffold that supported mesenchymal stem cell adhesion and growth. In another study, Kim et al. (2020) developed a chemically modified dECM using a methacrylate process for the extrusion of cell-laden skeletal muscle constructs that offer structural stability.

## 3D Biofabrication Technologies in Skeletal Muscle Engineering

The recent biofabrication technologies that enabled recapitulation of the skeletal muscle physiology in terms of uniaxial cell orientation and bundle-like fiber organization are discussed in this section with a focus on the benefits of 3D bioprinting technology. In particular, 3D bioprinting technology facilitates the layer-by-layer deposition of cell-laden bioinks in desired dimension under the optimized ink composition and post-printing crosslinking steps, which endows the engineered constructs with more physiologically relevant dimensions. In addition, cells or larger units such as cell aggregates, cell sheets, and cell-laden modules can be placed and assembled using the 3D bioprinting technology in a native organ-resembling spatial arrangement to maximize the cell–cell interaction.

### Mimicking Uniaxial Cell Alignment

**FIGURE 1:**
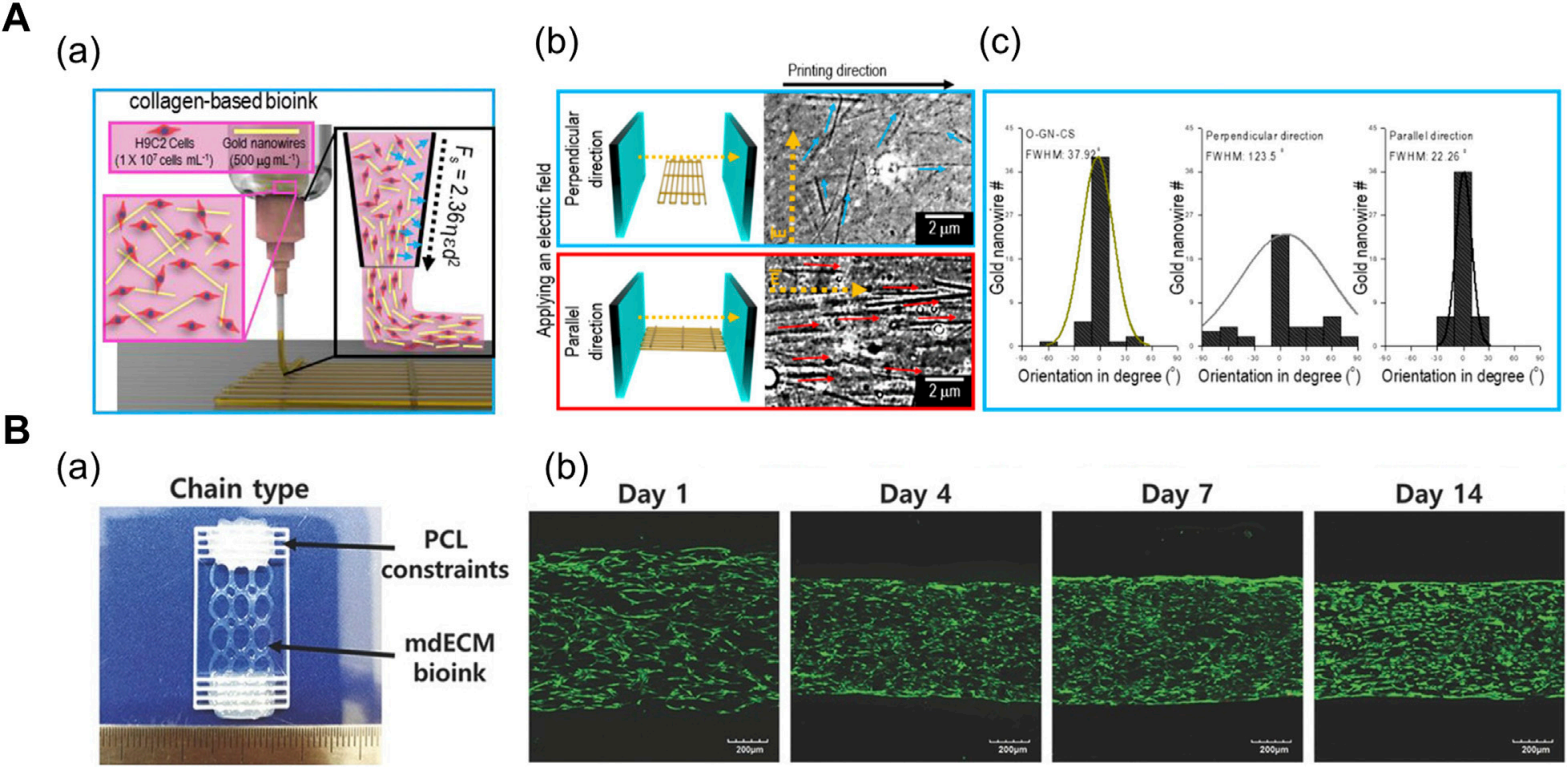
**(A)** Shear force-mediated cell alignment method. (a) Schematic of cell alignment during the extrusion printing process with gold nanowires aligned along the printing direction. (b) Electric field applied to promote the alignment of muscle cells through the alignment of gold nanowires. The optical images display the spatial distribution of gold nanowires under the electric field in directions perpendicular and parallel to the printing direction. (c) Distribution of orientation of gold nanowires under three conditions: no applied electric field (left), electric field applied in directions perpendicular (middle) and parallel (right) to the printing direction. Reprinted with permission from (Kim et al., 2019). **(B)** 3D-printed anchor structure for applying tensile force. (a) 3D-printed muscle construct comprising geometric constraints and muscle cell-laden mdECM bioink in chain shape. (b) Live/dead staining of printed muscle constructs at Days 1, 4, 7, and 14 of culture, revealing high cell viability (>90%) with minimal cell death. Construct compaction is accompanied by the longitudinal alignment of cells. Reprinted with permission from (Choi et al., 2016).

In general, uniaxial cell alignment is a representative morphological feature of the skeletal muscles that can be induced by applying shear (Mozetic et al., 2017; Kim and Kim et al., 2019; Kim et al., 2019) and tensile force (Choi et al., 2016; Agrawal et al., 2017; Cvetkovic et al., 2017; Das et al., 2019; Kong et al., 2021). The shear flow inside a cylindrical nozzle induces shear-thinning of the polymer solution. The wall shear forces exerted on the shear-thinning polymer induce the alignment of inherent polymer molecules (Compton and Lewis, 2014; Schwab et al., 2020). In particular, elongational forces are produced in the stretching region of the extruded polymer that can further accelerate the alignment and Kim et al. (2019) studied the shear-induced cell-laden collagen alignment and demonstrated that a fibrillating buffer solution (KCl and l-glycine) incorporated in the bioink induces the formation of collagen fibril bundle, closely mimicking its native structure (Kim and Kim et al., 2019). Based on their previous research, Kim et al. (2019) included gold nanowire as a nanofiller that was stably aligned during the extrusion process and strongly aligned upon electric field stimulation (Figure 1A). In addition, the orientation of the distribution of nanowires were manipulated under varying volume flow rates and nozzle speeds (Kim et al., 2019).

In order to apply tensile force, the muscle cell-laden hydrogels are confined around the two micropillars or posts. Upon tissue maturation and cell-mediated gel compaction, the hydrogel remain anchored to the posts and the cells align along the longest axis of the system. Choi et al. (2016) induced uniaxial muscle cell alignment by depositing polycaprolactone (PCL) anchors as a geometric constraint at both ends of the muscle construct (Figure 1B). The PCL and cell-containing mdECM bioink were 3D printed using an integrated composite tissue/organ building system (Choi et al., 2016). Alternatively, Agrawal et al. (2017) fabricated a hydrogel pillar using a photopatterning method. After injecting the hydrogel precursor solution into an inlet of the microfluidic device, a prefabricated photomask was positioned on the bottom of the device, followed by exposure to ultraviolet (UV) radiation, which resulted in the formation of two circular pillars. Thereafter, the muscle cell-laden gelatin methacrylate solution was injected into the device for photopolymerization, which created patterns between the cells and around the pillars (Agrawal et al., 2017).

### Mimicking Muscle Bundle Structure

In native skeletal muscle, a single fascicle comprises multiple muscle fibers arranged in a bundled form. Several biofabrication techniques have attempted to mimic the muscle bundle-like structures for recapitulating the hierarchical arrangement ranging from a single muscle fiber to a fascicle. This physiological aspect can be achieved as follows: fabrication of bundle-shaped 3D scaffold (Kim et al., 2018a; Kim et al., 2020), 3D bioprinting cell-laden construct with built-in channels (Miller et al., 2012; Kang et al., 2016; Kolesky et al., 2016; Kim et al., 2018b), and 3D embedded printing (Bertassoni et al., 2014; Choi et al., 2019; Skylar-Scott et al., 2019; Gao et al., 2021).

**FIGURE 2:**
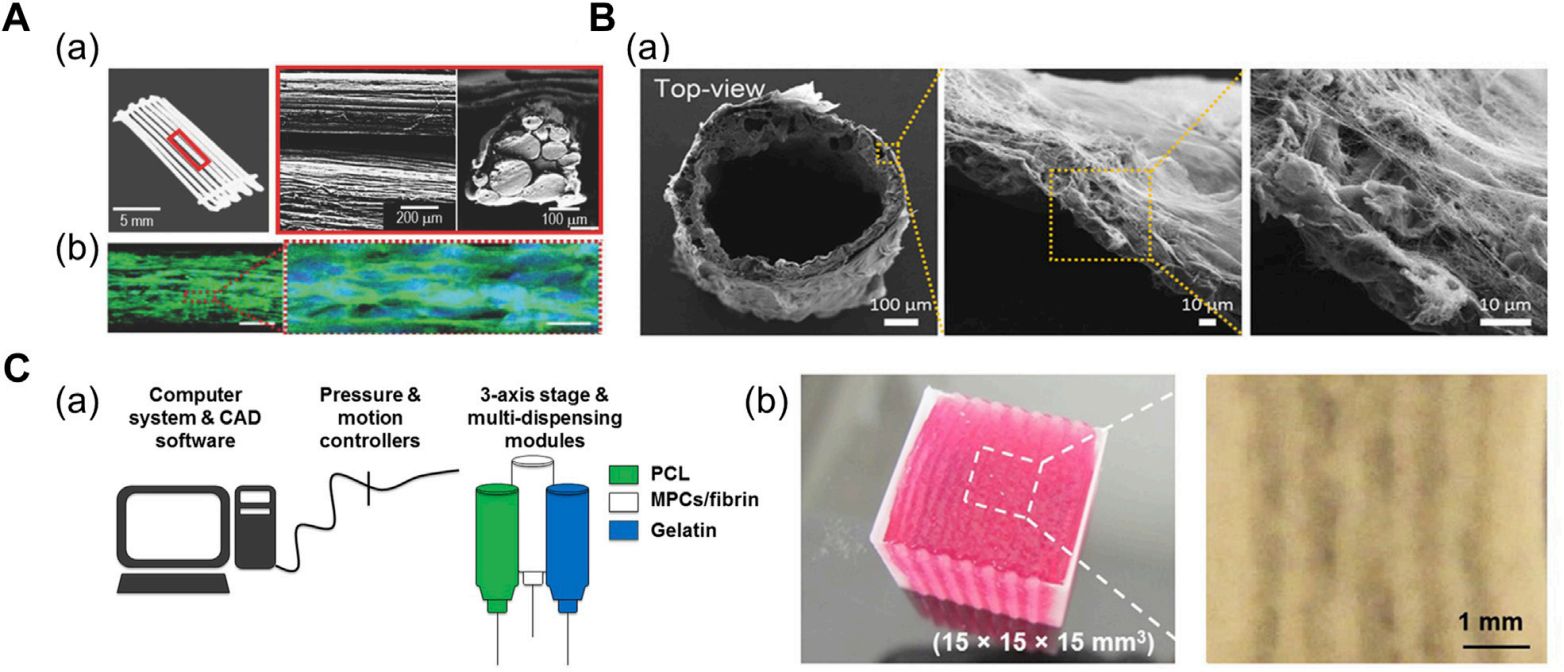
**(A)** Fabrication of PCL strut-based 3D scaffold. (a) Optical and scanning electron microscopy (SEM) images of micropatterned PCL scaffold. (b) Confocal images of cells at Day 14 immunostained for myosin heavy chain (green) and nuclei (blue), indicating the myogenic differentiation induced on the micropatterned PCL scaffold. Reprinted with permission from (Kim et al., 2018a). **(B)** Fabrication of thermally drawn PCL-fiber based 3D scaffold. (a) SEM images of PCL fiber-based scaffold after dECM absorption on scaffold surface. Reprinted with permission from (Jin et al., 2021). **(C)** 3D bioprinted cell-laden construct with built-in channels. (a) Schematic of integrated tissue-organ printing system. (b) 3D bioprinted multilayered muscle construct at scale: 15 × 15 × 15 cm. Microchannels are formed inside the construct after removal of gelatin patterns. Reprinted with permission from (Kim et al., 2018b).

A 3D scaffold comprising multiple polymeric struts or fibers adjacent to each other has been fabricated to mimic the bundle structure. Kim et al. (2018a) demonstrated shear-induced poly (vinyl alcohol) (PVA) alignment during the printing of PVA/PCL mixture (Figure 2A). Subsequent leaching of PVA yielded aligned micropatterns on the surface of the PCL scaffold, which was seeded with muscle cell-laden collagen solution (Kim et al., 2018a). Alternatively, Jin et al. (2021) fabricated the thermally drawn PCL fiber-based microchannel scaffolds (Figure 2B). After the scaffolds were soaked in the skeletal muscle dECM/muscle cell mixture and gelated at 37°C, the nuclear elongation was observed along the orientation of fibers, indicating the cell-guiding effect of the aligned fiber structures. In addition, the highly porous scaffold surface obtained from the salt-leaching method allowed the absorption of the dECM pre-gel solution into the scaffolds, which was evident from the presence of collagen mesh-like structures on the inner and outer sides of the fiber (Jin et al., 2021).

Microchannel-guided formation of aligned units has been utilized in a broad range of target tissues, including bone, nervous tissues, and vasculatures (Daly et al., 2018; Huang et al., 2018; Lee et al., 2020; Hwangbo et al., 2021). Sacrificial hydrogels—deposited as a temporary structure for structural support or generation of certain structures—are often removed by variations in temperature once the crosslinking of the engineered construct is complete; therefore, they have been widely adopted in 3D bioprinting for establishing built-in channels. In addition, thermoresponsive sacrificial polymers (i.e., gelatin and Pluronic F-127 (PF-127)) are printed as a temporary structure that dissolve in response to temperature variations after the bulk hydrogel has been crosslinked. Kang et al. (2016) printed supporting PCL pillars and sacrificial hydrogel patterns between cell-laden hydrogel patterns to yield a muscle bundle-like structure. Moreover, stretching and aligning muscle cells were observed inside these bundle structures 3 days after printing structure (Kang et al., 2016). Subsequently, Kim et al. (2018b) developed a larger construct (mm^3^–cm^3^) with multilayered muscle bundles to utilize cells from human biopsies and assess if the construct was applicable for severe muscle defect replacement (Figure 2C; Kim et al., 2018b).

Furthermore, 3D embedded printing utilizes a recently developed printing method named freeform reversible embedding of suspended hydrogel (FRESH). The major limitation of soft biomaterials such as ECM hydrogels is attributed to their poor shape-retention during the printing process that renders the desired resolution and 3D geometries unachievable (Hinton et al., 2015). The thermoreversible support bath prevents the collapse of the printed hydrogels and is subsequently melted away after the printing process. Moreover, complex structures such as branched tubular networks and trileaflet heart valves have been fabricated using the FRESH method (Bhattacharjee et al., 2015; Lee et al., 2019). Based on this method, Choi et al. (2019) directly printed a bundle-like structure in a granule-based support bath. Initially, they prepared the support bath by blending an 8% (w/v) gelatin solution to generate gelatin granules. Thereafter, mdECM struts were directly printed into the support bath with horizontal and vertical distances of 200 µm from each other. Subsequently, PVA was added into the gelatin granule to induce rapid polymerization of mdECM bioink during its extrusion (Choi et al., 2019).

## Maturation of 3D Tissue-Engineered Muscle

The development of 3D-engineered muscles results in physiologically mimetic tissues that can reflect the phenotypes that remain undiscovered or neglected in conventional systems. In this section, we discuss the recent biofabrication technologies for vascularization and innervation. Skeletal muscle vasculature participates in specific interactions with myofibers, which aid in muscle maturation and homeostasis. In particular, vascular endothelial growth factor (VEGF) stimulates the Akt pathway of muscle cells and induces myofiber hypertrophy in an autocrine manner (Yin et al., 2013). Long-term denervation or depletion of motor neuron innervation prohibits satellite cells from entering the mitotic cell cycle and leads to progressive myofiber atrophy (Kuschel et al., 1999). In addition, we review the studies that utilized electrical and mechanical stimuli for the maturation of 3D-engineered muscles.

### Vascularization

To date, several vascularization strategies have been developed to recapitulate the native vascular function (i.e., nutrient and oxygen supply to the highly oxygen-consuming organ) in the 3D-engineered muscle. The coaxial printing method and direct ink-writing method have been studied for the vascularization of engineered muscle as well as engineered tissues of other types.

Among the conventional vascularization methods, the hydrogel mold-casting method can generate hollow channel structures that are subsequently seeded with endothelial cells (ECs), forming endothelialized lumen structures (Bersini et al., 2018; Osaki et al., 2018; Redd et al., 2019). Osaki et al. (2018) observed emergent angiogenic sprouting from the surface of the endothelialized channels towards the muscle bundle between two channels, which mimicked myokine-regulated muscle angiogenesis. However, this method requires sequential stereolithography-based steps for obtaining the micropatterned mold. The multiple channel structures should be individually obtained to allow active diffusion of nutrients, thereby adding complexity to the mold-preparation step.

**FIGURE 3:**
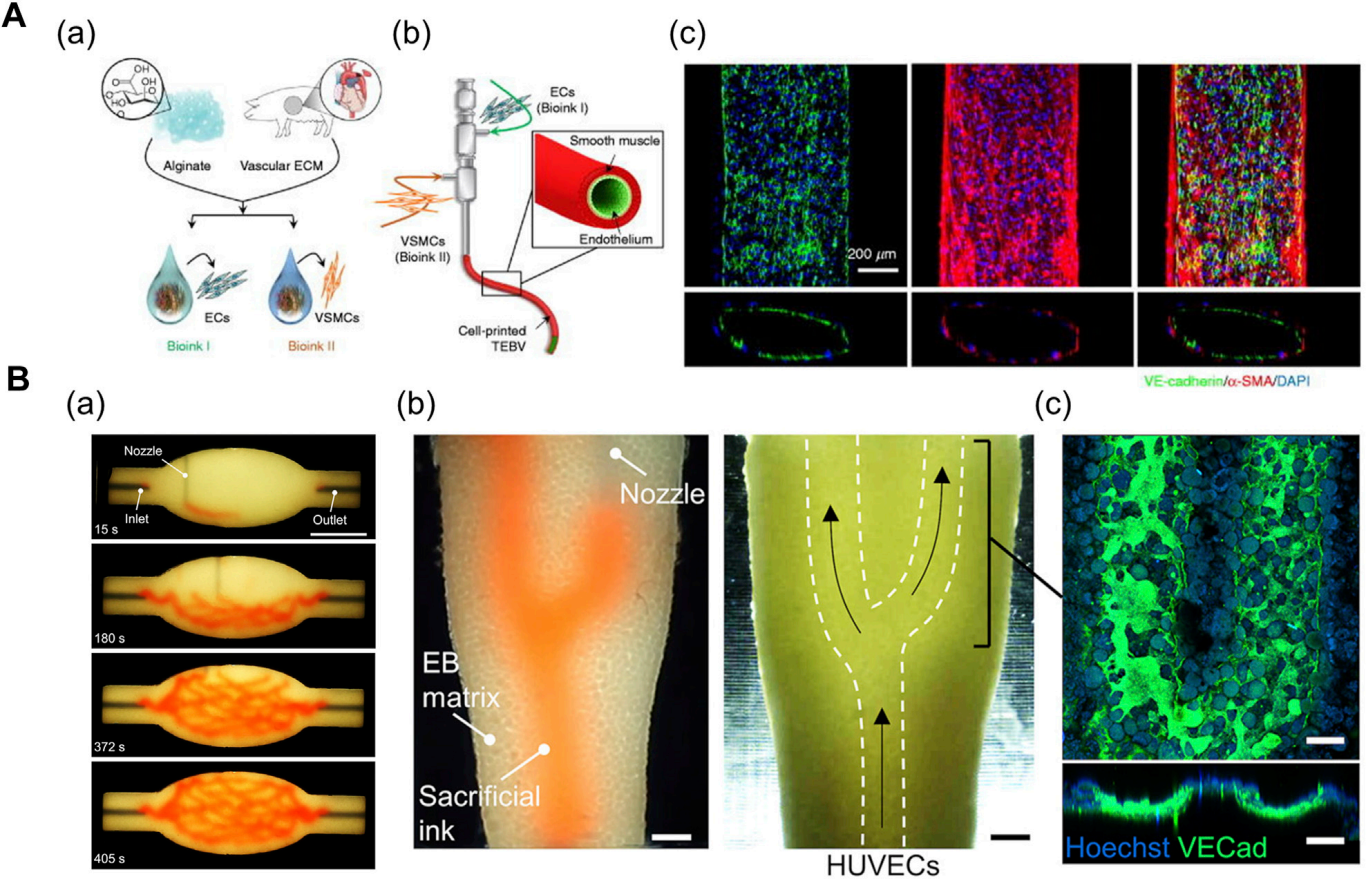
**(A)** Coaxial printing method. (a) Formulation of vascular tissue-derived bioinks for promoting vascular maturation. (b) Schematic of triple-coaxial cell printing method yielding compartmentalized vascular structure. (c) Confocal image of printed vascular structure with combined expression of endothelial and muscular markers. Reprinted with permission from (Gao et al., 2019). **(B)** Direct ink-writing method. (a) Sequence of branched and hierarchical vascular network fabricated with direct ink-writing method. (b) Perfusion of ECs into evacuated channel (white-dashed lines). (c) Expression of VE-cadherin after 20 h of perfusion displays formation of endothelium monolayer. Reprinted with permission from (Skylar-Scott et al., 2019).

In general, 3D printing enables the iterative one-step fabrication of composite structures. For instance, the coaxial printing method enables direct fabrication of two- (Zhang et al., 2016; Gao et al., 2017; Choi et al., 2019) or three- (Gao et al., 2019; Gao et al., 2021) layered 3D conduits of varying geometries and dimensions (Figure 3A). Zhang et al. (2016) bioprinted a 3D microfibrous scaffold structure using a composite bioink comprising alginate (GelMA) and photoinitiator. The alginate in the core part of the nozzle was exposed to a chemical crosslinking agent (Ca^2+^) provided from the shell part of the nozzle during the printing process. The printed scaffold manifested mechanical stability after the UV crosslinking of GelMA. Ultimately, the cardiomyocytes were seeded onto the stiffened microfibrous scaffold after the bioprinted ECs self-assembled into a layer of confluent endothelium, thereby resembling the structure of native myocardium (Zhang et al., 2016). In another research, Choi et al. (2019) fabricated a compartmentalized core–shell structure in which a layer of ECs surrounded the muscle fiber-like struts, thereby closely mimicking the hierarchical architecture of vascularized muscle. Within a coaxial nozzle, the syringe containing the muscle cell-laden mdECM bioink and the syringe containing the EC-laden vascular dECM bioink were attached to the core and shell parts, respectively. In comparison to the muscle-only construct, the higher twitch and tetanic peak forces of the vascularized muscle confirmed its enhanced maturity (Choi et al., 2019).

The direct ink writing method proposed by Skylar-Scott et al. (2019) enables the fabrication of complex vasculature in a cell-filled support bath via 3D embedded printing (Figure 3B). This method is novel owing to the high cellular density of the support bath (up to 10^8^ cells/ml) (i.e., living tissue matrix) which is the prerequisite for physiologically relevant tissue models. The living tissue matrix exhibited a self-healing, viscoplastic behavior that allowed the printing of sacrificial ink in the desired network structure without crevasses formation on the printing path. Subsequently, the temperature-mediated melting of sacrificial gelatin ink and the simultaneous stiffening of the matrix yielded hollow vasculature networks, followed by the perfusion of EC suspension (Skylar-Scott et al., 2019). Based on a similar approach, Neufeld et al. (2021) fabricated a 3D glioblastoma model with a perfusable vasculature. Initially, the PF-127-based vascular bioink was deposited on the surface of cancer cell-laden fibrin bioink, followed by casting another layer of fibrin bioink onto the vasculature. Once the fibrin bioink achieved the desired physiological stiffness, the PF-127 was washed out with cold PBS, and a mixture of ECs and pericytes were injected into the vascular channels (Neufeld et al., 2021). Overall, the direct ink-writing method allows freeform fabrication of tissue-specific vascular structures. However, the mechanical properties of the support bath inks and vascular inks should be carefully manipulated in combination to achieve the desired geometry of vasculatures.

### Innervation

Motor neuron innervates the skeletal muscle via neuron–myofiber contacts or neuromuscular junctions (NMJs), which mediate the conversion of neuronal signal into muscle contraction. Functional NMJs have been engineered by multiple biofabrication methods including 3D co-culture (Morimoto et al., 2013; Bakooshli et al., 2019) of neuron and myofibers, and 3D bioprinting of structures with a spatial organization that induces spontaneous neurite formation (Cvetkovic et al., 2017; Kim et al., 2020; Kong et al., 2021).

**FIGURE 4:**
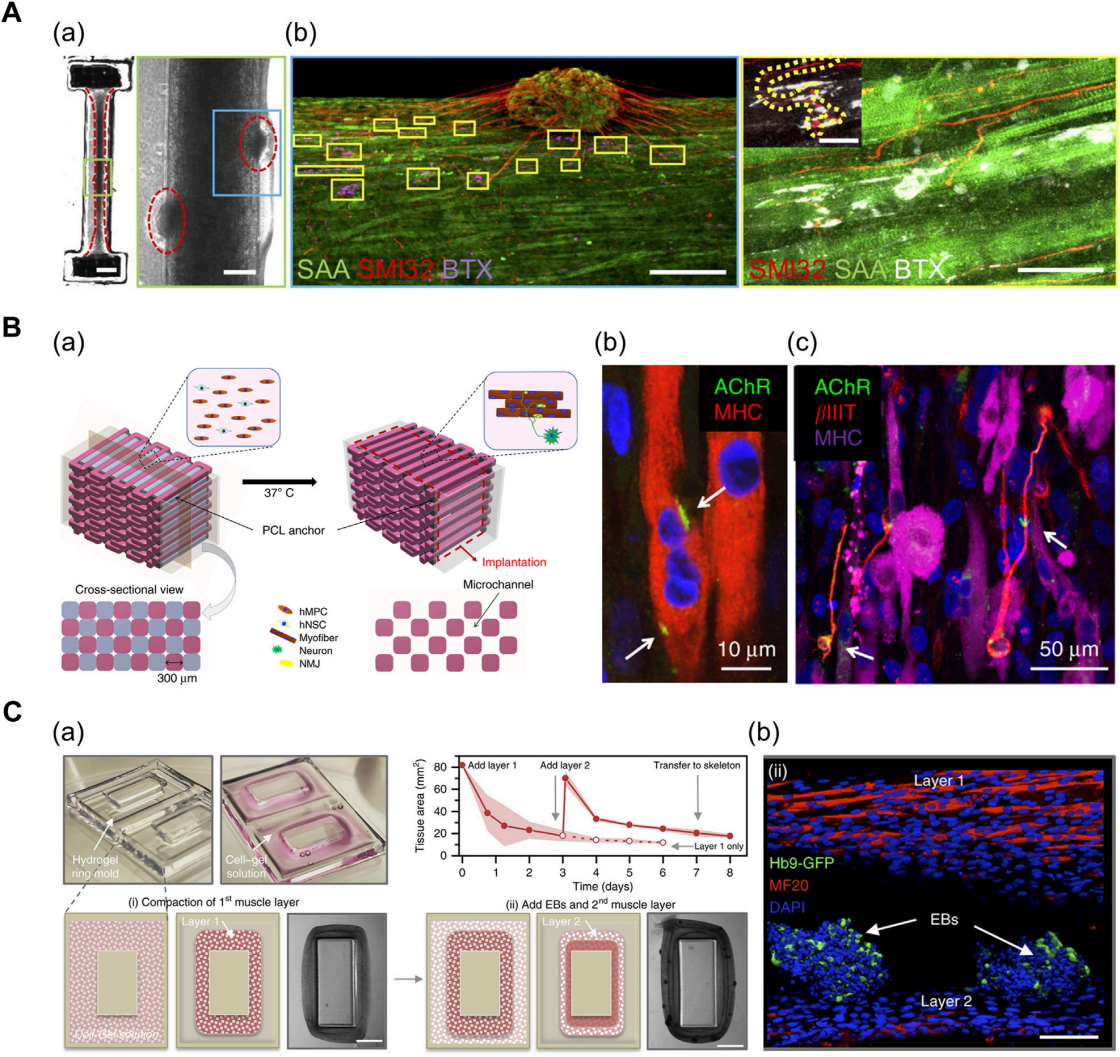
**(A)** 3D muscle–motor neuron co-culture. (a) phase contrast image portraying differentiated myofiber co-cultured with motor neuron spheroid for 2 weeks. (b) Confocal image displaying co-localization of neurite terminals (red) and acetylcholine clusters (magenta/white) on a striated myofiber (green). SAA: sarcomeric α-actinin, SMI32: anti-200 kD neurofilament heavy antibody, BTX: α-bungarotoxin. Reprinted with permission from (Bakooshli et al., 2019). **(B)** 3D bioprinted innervated muscle construct. (a) 3D CAD modeling of bioprinted construct. Hollow microchannels formed after removing sacrificial patterns. (b) Confocal image of acetylcholine receptors (green) prepatterned on myofibers (red). (c) Confocal image of neurites (red) contacting acetylcholine receptor clusters (green). βIIIT: beta-III tubulin. Reprinted with permission from (Kim et al., 2020). **(C)** Modular muscle–motor neuron co-culture system. (a) Schematic of fabrication of multilayered tissue ring. The cross-sectional tissue area decreased over time as cell–gel solution compacted around the mold. (b) Confocal image indicating differentiated myofibers (red) and motor neuron EBs (green). Hb9: post-mitotic MN-specific Hb9 promoter. MF20: anti-myosin heavy-chain antibody. Reprinted with permission from (Cvetkovic et al., 2017).

In particular, the neurons can self-organize to form NMJs in the 3D co-culture of motor neurons (MNs) and muscle cells. Multiple biofabrication skills, including the usage of patterned stamps and molds, facilitate the integration of MN spheroids with differentiated myofibers. Morimoto et al. (2013) used polydimethylsiloxane (PDMS) stamps for patterning Matrigel to form aligned muscle bundle, and subsequently, added mouse neural stem cell-derived spheroids (mNSCs) onto the muscle bundle. The mNSCs were successfully differentiated into motor neurons, and acetylcholine receptors were formed at the muscle bundle-spheroid contacts. Moreover, the muscle bundle contracted unidirectionally in response to glutamic acid activation of neurons, indicating the formation of functional NMJs (Morimoto et al., 2013). Based on a similar approach, Bakooshli et al. (2019) prepared a dumbbell-shaped PDMS mold that was seeded with a mixture of human pluripotent cell-derived MN spheroids and human muscle progenitor cells suspended in fibrin-based hydrogel (Figure 4A; Bakooshli et al., 2019). The expression of NMJ-localized protein, such as synaptic MuSK protein, confirmed the recapitulation of early NMJ synaptogenesis in this model.

In addition, 3D bioprinting technology facilitates the precise positioning of multiple cell types in a desired organization and array. Kim et al. (2020a) generated a functional NMJ inside a 3D-printed multilayered bundle-shaped construct containing a hollow microchannel structure (Figure 4B). Fibrinogen/gelatin/hyaluronic acid/glycerol bioink containing human muscle progenitor cells and human neural stem cells (hNSCs), acellular sacrificial bioink, and a supporting PCL pillar were deposited using multidispensing modules. Consequently, βIIIT^+^ neurites contacting the acetylcholine receptor clusters were formed on the myofibers, which confirm the formation of NMJs (Kim et al., 2020). In an alternative approach, Cvetkovic et al. (2017) used a stereolithography-based 3D-printing technique to fabricate a modular co-culture system (Figure 4C). A 3D tissue ring, previously formed by integrating two layers comprising differentiated myofibers and motor neuron embryoid bodies (EBs) in a 3D-printed ring mold, was transferred to the hydrogel skeleton to mimic the physiological muscle–tendon–bone arrangement. This co-culture arrangement exhibited the extension of the neurite toward the differentiated muscle and the formation of the acetylcholine receptor cluster (Cvetkovic et al., 2017).

### Contractility

Contractility is one of the most widely accepted muscle functional readouts for distinguishing between normal and diseased muscle phenotypes (Sharples et al., 2012; Juhas et al., 2018). In context, the early detachment of spontaneously contracting myofibers allowed only partial contractility analysis in 2D cultures, which were confined to the variations in related gene expressions (Nikolić et al., 2012; Tarum et al., 2017). Various external stimuli have been applied to improve the contractility of the 3D-engineered muscle. These stimuli aim to mimic the native stimuli the developing skeletal muscle receives (Westerga and Gramsbergen, 1994; Eken et al., 2008), which induce muscle hypertrophy. In general, electrical stimulation and mechanical loading are frequently utilized in combination.

First, electrical stimulation is applied as a surrogate of neural input that are delivered to individual myofibers to induce muscular contraction. In addition, the regenerating muscles in the self-repair process require electrical signals from motor neurons, implying that the electrical stimulation can enhance the function of the engineered muscle (Kim et al., 2019). Upon applying the electrical stimulation, the pulse energy and width should be optimized to minimize the tissue damage (Khodabukus and Baar, 2012). Grosberg et al. (2011) applied an electrical field stimulation for cantilever-type thin muscular films and measured the transmembrane potential of cardiomyocytes. This revealed an action potential morphology, indicating the contractility achieved in the tissue (Grosberg et al., 2011). Additionally, Khodabukus et al. (2019) demonstrated that a 3D-engineered muscle electrically stimulated with 1 and 10 Hz of frequency for 1 week resulted in an increased contractile force (nonstimulated: 9.1 ± 0.38 mN mm^−2^, 1 Hz: 19.3 ± 0.63 mN mm^−2^, 10 Hz: 18.9 ± 0.69 mN mm^−2^). More importantly, the stimulation of 1 Hz enabled the highest specific force generation among the outputs of engineered human muscle reported to date (Khodabukus et al., 2019).

On the other hand, mechanical loading mimics the consistent passive stretch exerted upon the embryonic muscle. Kim et al. (2019) reported that combined electrical and mechanical stimulation induced the remodelling of ECM network structures in engineered muscle tissue, such that the force transmission was facilitated without impeding muscle contraction. In particular, 20 min of alternating electrical and mechanical stimulations resulted in 31% higher contractile force (Kim et al., 2019). Moreover, Aguilar-Agon et al. (2019) studied the influences of mechanical loading on anabolic shift based on the results of *in vivo* resistance exercise regimens. Insulin-like growth factor-1, which is associated in skeletal muscle growth, was upregulated 21 h after the cessation of mechanical load (15% elongation of engineered construct); after 45 h from cessation, the atrophic gene MAFbx underwent significant downregulation. In addition, maximal isometric force exhibited an increase of 140% (21 h) and 265% (45 h) as compared to nonloaded constructs (Aguilar-Agon et al., 2019). Collectively, the electrical stimulation and mechanical loading could result in significant muscle hypertrophy and increase in force generation, which can potentially enable the maturation of engineered muscle to a similar extent as that in human adult muscle.

## Recapitulation of Skeletal Muscle Tissue-Related Diseases

The representation of a more human-like pathology for preclinical studies relies on the maturity and complexity of the model. In context, several muscle-disease phenotypes have been achieved in 3D-engineered skeletal muscles, which were not earlier displayed in 2D cultures or animal models. The recent studies conducted using a 3D-engineered muscle model that recapitulated the major disease phenotypes of severe muscular dystrophies (MDs) and inflammatory muscle diseases are discussed and reviewed herein.

### Muscular Dystrophies and Muscle Weakness

Muscular dystrophies arise from mutations in genes encoding various proteins such as muscle ECM, cytoplasmic and plasma membrane proteins that result in progressive muscle weakness or wasting (Tedesco and Cossu, 2012). The most common and severe type of dystrophy, DMD, is an X-linked recessive disorder that occurs almost exclusively in males. The underlying mechanism of DMD is attributed to the mutation in the gene encoding dystrophin, a membrane-bound cytoskeletal protein that links the intracellular actin cytoskeleton to extracellular matrix. Therefore, the absence of dystrophin destabilizes the membranes and causes consistent muscle fiber damage during contraction, which results in consistent fiber degeneration (Nowak and Davies, 2004; Allen and Whitehead, 2010).

**FIGURE 5:**
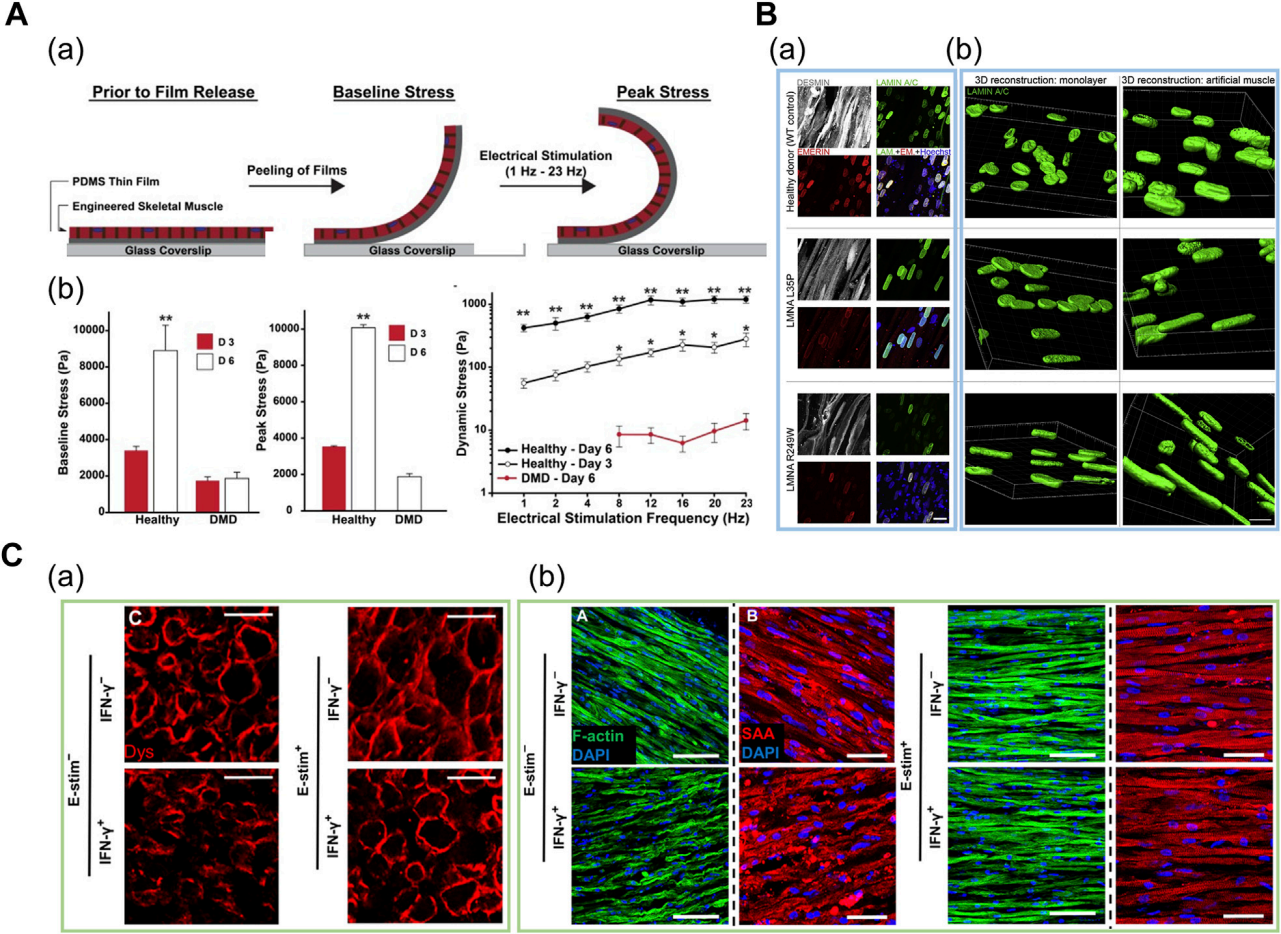
**(A)** Engineered human DMD model in cantilevered design. (a) Schematic of tissues undergoing baseline and peak stress. (b) Baseline, peak, and dynamic stress in healthy tissues at day 3 and day 6, and in DMD tissues at day 6. Reprinted with permission from (Nesmith et al., 2016). **(B)** Patient-derived iPSC-based engineered LMNA model. (a) Whole-mount confocal images of myotubes (gray) and nuclear lamina (green for LAMIN A/C and red for emerin) on iPSC-based engineered muscles derived from healthy and two LMNA mutants. (b) 3D nuclear reconstructions of patient-derived iPSCs differentiated in 3D-engineered muscles highlight more prominent nuclear deformities and elongation as compared to monolayer cultures. Reprinted with permission from (Maffioletti et al., 2018). **(C)** Engineered human cardiac muscle for modeling exercise-mediated attenuation of IFN-γ-induced muscle wasting. (a) Cross-sections of engineered muscle immunostained for dystrophin indicates attenuated muscle atrophy under exercise–mimetic electrical stimulation. (b) Longitudinal sections of engineered muscle immunostained for F-actin (green) and sarcomeric α-actinin (red). Electrical stimulation prevented IFN-γ-induced decrease of sarcomere organization. Reprinted with permission from (Chen et al., 2021).

Thus, patient-derived cells are cultured under uniaxial tension or micropatterned substrates to form a 3D myofiber for modeling DMD. During and after the culture, the cells are screened for degenerative morphologies such as less sarcomeric α-actinin organization and decreased contractile force. Nesmith et al. (2016) fabricated a thin, contractile muscular film composed of DMD patient-derived cells in a cantilevered design by printing an array of thin bands using a microcontact printing method (Figure 5A). Under electrical stimulation with increasing frequencies ranging from 1 to 4 Hz, the dynamic stress on the healthy tissues on day 6 was at least one order of magnitude higher in comparison to the diseased tissues, as evaluated based on the variations in the radius of film curvature. The results implied that the impaired force transmission caused by the absence of dystrophin was recapitulated in this system (Nesmith et al., 2016). Subsequently, Khodabukus et al. (2019) used fibrin-based 3D “myobundles” confined to rectangular Teflon frames to demonstrate the correct membrane-bound localization of dystrophin. Moreover, the electrical stimulation increased the dystrophin, myosin heavy chain, and sarcomeric α-actinin content (Khodabukus et al., 2019). Overall, the 3D-engineered muscle models can represent more adult-like phenotypes as compared to the developmentally less matured cytoplasmic localization of dystrophin in conventional 2D culture (Serena et al., 2016).

Maffioletti et al. (2018) applied a tensile force to fibrin-based 3D engineered tissue and generated a multilineage patient-specific model using a patient-derived iPSC of laminopathy (LMNA), which is one of the MD types (Figure 5B). LMNA results from the mutation of the LAMIN A/C gene encoding a protein that composes the nuclear lamina. Under uniaxial tension, hiPSCs were successfully differentiated into myofiber-resembling structures. Notably, the genotype–phenotype correlation was achieved as hiPSCs displayed significant nuclear abnormalities such as nuclear elongation, which was absent in normal muscles and other dystrophic hiPSC lines (Maffioletti et al., 2018). Moreover, Bersini et al. (2018) demonstrated a multicellular DMD model composed of cells from human biopsy to mimic the aberrant fibrosis. They fabricated a spatially organized mesoscale system where differentiated myofibers were surrounded by 3D microvascular network and a fibroblast layer. In particular, collagen and fibronectin secretion levels were significantly higher in patient-derived fibroblasts as compared to normal and TGF-β1 treated fibroblasts. This trend was not displayed in 2D triculture system, indicating that the 3D mesoscale system supported more physiologically relevant fibrosis progression such as muscle-specific fibroblast recruitment. Furthermore, the α-smooth muscle actin, which is expressed in the fibroblasts residing in the dystrophic muscle, was expressed in high levels in the DMD fibroblasts, thereby indicating an accurate representation of the DMD-affected tissue microenvironment (Bersini et al., 2018).

### Inflammatory Muscle Disease and Skeletal Muscle Self-Repair

Skeletal muscle self-repair in response to mild injuries is modulated by the interaction between the muscle-resident satellite cells and the immune system via the process of satellite cell activation, proliferation, and differentiation. However, excessive and unregulated inflammatory responses are associated with muscle loss and weakness, which has been reported in several types of diseases including sarcopenia, cachexia, chronic obstructive pulmonary disease and rheumatoid arthritis. In particular, several clinical studies have reported that elevated IFN-γ levels in autoimmune diseases and chronic inflammation contribute toward muscle wasting.

Based on a 3D fibrin-based engineered cardiac muscle tissue composed of human primary cells, Chen et al. (2021) assessed the influence of exercise on IFN-γ-induced muscle wasting (Figure 5C). In this model, muscle atrophy was characterized by weaker and slower muscle contraction and reduced calcium-handling protein expression. Notably, the exercise-mimetic electrical stimulation with IFN-γ treatment rescued the sarcomeric protein organization and elongated the myotube, thereby implying an attenuated muscle atrophy condition. In particular, inhibited expression of IFN-γ-induced JAK (Janus kinase)/STAT (signal transducer and activator of transcription) pathway downstream proteins suggested the previously unknown, exercise-mediated cell autonomous anti-inflammatory response that occurs in the muscle tissue, besides the paracrine signalling which has been traditionally suggested as the fundamental mechanism. Furthermore, prevention of muscle atrophy after treatment of FDA-approved small-molecule inhibitors of JAK/STAT pathway confirmed the utility of this platform for preclinical drug screening (Chen et al., 2021).

Juhas et al. (2018) established a 3D co-culture system based on previously referred 3D engineered muscle to assess the role of macrophages in skeletal muscle regeneration process. An inhibited endogenous self-repair process was successfully mimicked as progressive myofiber degeneration was induced after cardiotoxic injury, which resembled a severe muscle injury. Then a robust regenerative response was induced upon incorporating bone-marrow derived macrophages with lower levels of activated caspase 3, which indicated that the anti-apoptotic paracrine signalling between the muscle and macrophages were mimicked in this model. Furthermore, the implantation of macrophage-incorporated engineered muscles in a mouse dorsal window-chamber model resulted in robust blood vessel ingrowth and functional muscle regeneration. As such, the *in vitro* incorporation of macrophages can be considered as an effective approach for preconditioning an implantable construct in a pro-regenerative milieu before implantation (Juhas et al., 2018).

## Future Applications

To date, the incorporation of patient-derived cells and induction of chemical injuries in 3D-engineered muscle models have successfully modeled multiple types of muscle diseases. Although several muscle diseases exhibit combined features of muscle and connective tissue pathology (e.g., limb-girdle muscular dystrophy) (Csapo et al., 2020), only certain models have utilized the aspect of the diseased ECM. Aberrant fibrosis accounts for one of the most prominent features of DMD (Bersini et al., 2018), as it causes the accumulation of collagen I and fibronectin. In particular, the combination of mechanical stimuli and diseased ECM could accurately model the progression of multiple type of unveiled muscular dystrophies. Moreover, 3D muscle-like architecture in engineered muscles might aid in the accurate recapitulation of the cell–matrix interaction.

**FIGURE 6:**
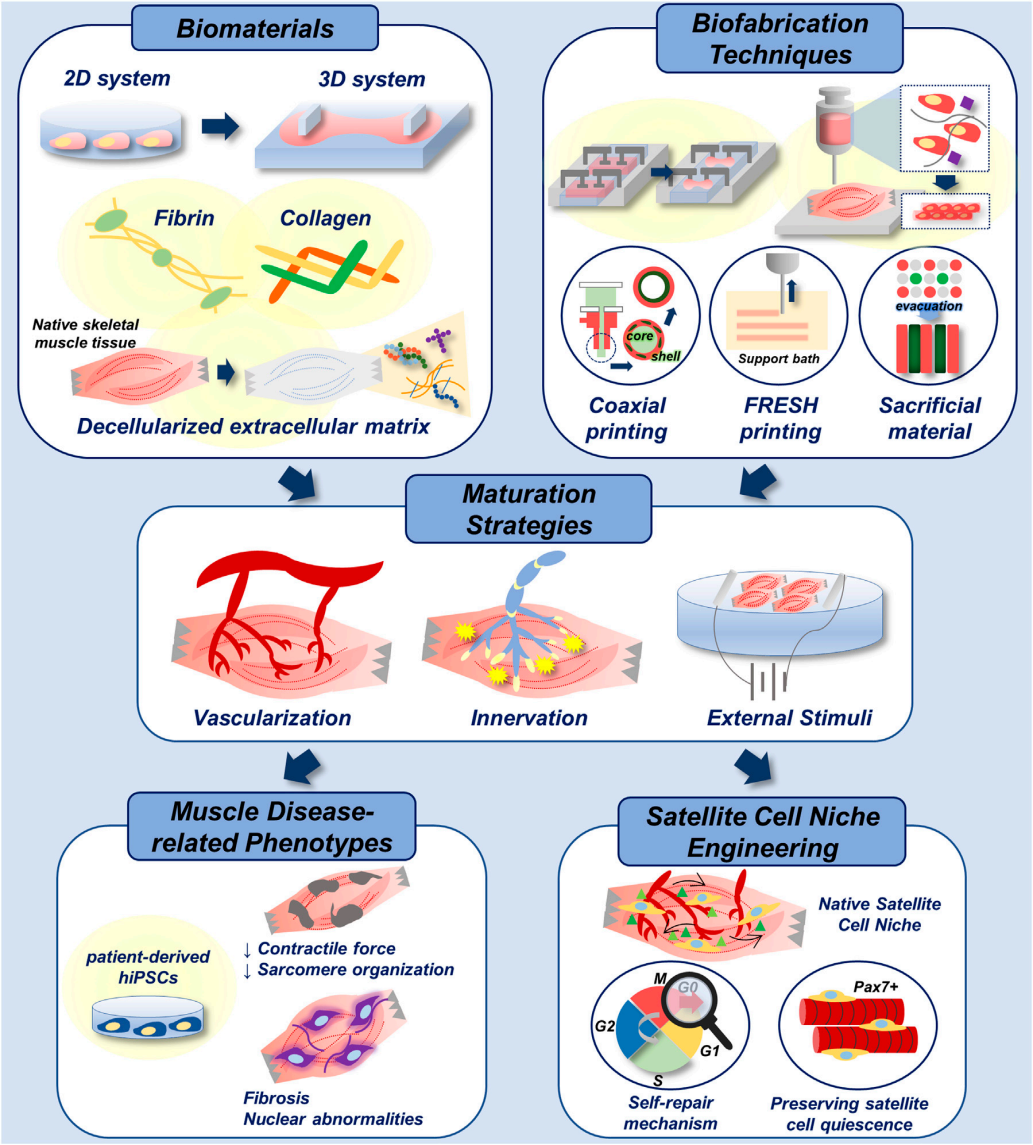
Biofabrication of skeletal muscle disease models. Multiple biomaterials and biofabrication techniques can be utilized to develop 3D-engineered muscle with native tissue-like stiffness and topography (top). Functional maturation of 3D-engineered tissue is achieved by vascularization, innervation and electrical/mechanical stimuli (middle). With the incorporation of patient-derived hiPSCs, addressed biofabrication techniques can be applied in recapitulation of muscle disease-related phenotypes *in vitro.* In addition, they could be applied in engineering of native satellite cell niche for the study of muscle self-repair mechanism, and for the preservation of satellite cell quiescence *in vitro*, which enhances the therapeutic efficacy of cells (bottom).

In addition, engineering a native satellite cell niche-mimicking microenvironment is an emerging topic. In an attempt to preserve satellite cell quiescence *in vitro*, Quarta et al. (2016) fabricated a 3D-engineered muscle composed of muscle satellite cells using a micropost platform. They screened for the micropost stiffness that was known to affect the stem cell behaviour. Moreover, they defined the factors that could assist satellite cell quiescence *in vitro* and applied it in the culture of the engineered muscle (Quarta et al., 2016). Alternatively, Lee et al. (2020) utilized a 3D microfluidic platform to establish vascular architecture of the satellite cell niche *in vitro*, where the intercirculation of niche factors between young and old satellite cells were demonstrated (Lee et al., 2020). These models are potentially useful for modelling the satellite cell activation, quiescence, self-renewal and differentiation (Ishii et al., 2018; Csapo et al., 2020) which all aid in skeletal muscle self-repair mechanism (Figure 6).

The preservation of satellite cell quiescence impacts the therapeutic efficacy of the cells as well. Ishii et al. (2018) identified a laminin-type component of basement membrane that encapsulates satellite cells. Thereafter, they prepared a recombinant protein that partially contained the C-terminal of the original basement membrane component, which was mixed with Matrigel. Under the *in vitro* culture of using the substrate, the satellite cell quiescence was preserved, and the enhanced muscle regeneration capacity was confirmed *in vivo* (Ishii et al., 2018). Overall, these approaches would endow isolated primary cells with enhanced engraftment potential and muscle regeneration capacities, which could facilitate the improvement of current regenerative therapies for volumetric muscle loss.

Endogenous muscle self-repair is supported by interactions between muscle satellite cells and multiple types of muscle-resident cells, including macrophages, CD3^+^ T cells, endothelial cells, and fibro-adipogenic progenitors that collectively form a complex inflammatory milieu (Fu et al., 2015; Quarta et al., 2017; Judson et al., 2020). However, current *in vitro* models of muscle self-repair partially recapitulate the microenvironment of the regenerating muscle, as satellite cells are co-cultured with either macrophages or endothelial cells (Juhas et al., 2018; Lee et al., 2020). Therefore, the incorporation of multiple cell types is required for an in-depth understanding of the mechanism of satellite cell quiescence in regenerating muscles. Moreover, the sorting and expansion of the desired cell population among a larger pool of cells remain challenges for obtaining a sufficient number of cells and in applying these models in preclinical settings.
